# Upregulated small GTPase immunity-associated proteins confer resistance to *Neospora caninum* in rat and bovine cells

**DOI:** 10.3389/fcimb.2025.1674380

**Published:** 2025-10-14

**Authors:** Abdur Rehman Azam, Md Mukthar Mia, Julie Bedwani, William H. Witola

**Affiliations:** ^1^ Department of Pathobiology, College of Veterinary Medicine, University of Illinois Urbana-Champaign, Urbana, IL, United States; ^2^ Department of Veterinary Clinical Medicine, College of Veterinary Medicine, University of Illinois Urbana-Champaign, Urbana, IL, United States

**Keywords:** *Neospora caninum*, infection resistance, GIMAPs, rat, bovine

## Abstract

**Background:**

Neosporosis is a leading cause of abortions and neonatal mortality resulting in significant global economic losses in cattle production, and is also a common cause of a fatal neuromuscular degenerative disease in dogs, for which there are no effective treatments nor prophylactics available. Elucidation of *Neospora*-specific mechanisms that resistant hosts employ to orchestrate defenses against the parasite could hold the key to unveiling novel strategies for developing effective control approaches against neosporosis. Previously, we reported that the Lewis rat resists intracellular *Toxoplasma gondii* growth by augmenting the expression of *G*TPase *Immunity*-Associated Proteins (GIMAPs), namely GIMAP 4, 5, and 6 that mediate the resistance phenotype.

**Methods:**

Herein, we investigated the effect of upregulated expression of GIMAPs on the growth and proliferation of *Neospora caninum* (an evolutionarily close relative to *T. gondii*) in rat and bovine cells. First, we used two rat strains (Lewis and Brown Norway rats) to determine the effect of *N. caninum* infection on GIMAPs expression, and the ability of parasite proliferation in the respective rat cells and tissues. We analyzed the effect of GIMAP 4, 5 and 6-induced upregulation in permissible rat and bovine macrophages on *N. caninum* growth, and determined the molecular networks engaged by GIMAPs in orchestrating intracellular parasite killing.

**Results:**

We found that, unlike the Brown Norway rat, the Lewis rat is refractory to *N. caninum* infection, with a concomitant augmentation of GIMAP 4, 5 and 6 expression in response to infection. Corroboratively, overexpression of GIMAP transgenes in a *N. caninum*-permissive rat macrophage cell line induced accumulation of LAMP 1 (lysosome marker protein) on the parasitophorous vacuole membrane (PVM), resulting in vacuole acidification and restriction of *N. caninum* proliferation. Further, we found that bovine GIMAP 4, 5 and 6 are orthologous to rat GIMAPs, with a conserved AIG1 domain. Intriguingly, overexpression of bovine GIMAP transgenes in a *N. caninum*-susceptible bovine macrophage cell line inhibited intracellular proliferation of the parasites.

**Discussion:**

Collectively, our findings imply that upregulation of GIMAP 4, 5, and 6 mediate robust refractoriness to *N. caninum* through induction of lysosomal fusion to the otherwise non-fusogenic PVM, resulting in vacuole acidification and destruction of intracellular parasites.

## 1 Introduction

*Neospora caninum* is an apicomplexan protozoan parasite that is closely related to *Toxoplasma gondii* (Reid et al., 2012; Athanasiou et al., 2021). *N. caninum* is capable of infecting a wide range of warm-blooded animals and is a leading cause of abortions and neonatal mortality in cattle, as well as a cause of fatal neuromuscular degeneration in dogs (Dubey et al., 2007; Kasap et al., 2020). Neosporosis is estimated to result in global livestock production losses of between US $43.08 billion and US $320.98 billion annually (Dubey et al., 2007; Reichel et al., 2020).

The complex life cycle of *N. caninum* involves definitive hosts (canids) that typically get infected by ingesting tissues from infected intermediate hosts (non-canids). The parasite undergoes sexual reproduction in the intestinal cells of canids, leading to the production of parasite oocysts that are shed in the animal feces and contaminate the environment (Reichel et al., 2020). Intermediate hosts, including cattle, sheep, goats, and other non-canids, get infected by ingesting the *N. caninum* oocysts in contaminated feed or water (Almería and López-Gatius, 2013). In both canids and non-canids, vertical transmission from an infected dam to the fetus can occur and is known to be predominant in cattle, allowing the parasite to persist within cattle herds for multiple generations (Dubey et al., 2007). In infected hosts, two distinct parasite stages exist, including the intracellular proliferative tachyzoite stage that is found in various tissues during the acute phase of the disease, while the dormant bradyzoite stage is found in cysts mainly in brain and muscle tissue during the chronic phase of the disease (Almería and López-Gatius, 2013; Winzer et al., 2020). Intracellularly, tachyzoites live and replicate inside the parasitophorous vacuole, a non-fusogenic membrane-bound compartment that protects them from host lysosomal degradation (Nolan et al., 2015).

Despite the significant economic losses attributed to neosporosis in the livestock industry, there is still no effective treatment nor vaccine available. Development of strategies for effective therapeutics and control of neosporosis will require, among others, identification and functional characterization of host effector molecules that are important in mediating host resistance to *N. caninum* infection. In our previous work focused on unraveling molecular factors that direct the early, robust, protective, innate immune responses against *T. gondii* in the Lewis rat (that is inherently refractory to toxoplasmosis), we identified GTPase Immunity-Associated Proteins (GIMAPs), namely, GIMAP 4, 5, and 6, that are upregulated in response to *T. gondii* infection and contribute to the host’s resistance phenotype (Kim et al., 2018). GIMAPs are recently described small GTPases that are conserved in mammals (Filén and Lahesmaa, 2010; Pascall et al., 2013). Their functions and molecular mechanisms in mammalian host innate immunity against intracellular pathogens are just beginning to be explored. GIMAP 4, 5, and 6 contain a conserved AIG1-type G (avrRpt2-induced gene) domain comprising a Walker A motif (G1 box), a Walker B motif (G3 box), and boxes G2, G4, and G5 that are consistent in small GTPases (Kim et al., 2018). The GTP-binding site motif A (P-loop within the G1 box) is required for oligomerization of effector molecules to the parasitophorous vacuole membrane (PVM) (Howard et al., 2011; Kim et al., 2018). GIMAP 4, 5, and 6 co-localize with *T. gondii* GRA5 (a PVM marker protein) and induce translocation of a lysosome marker protein (LAMP1) to the PVM, leading to inhibition of parasite growth (Kim et al., 2018). Herein, we endeavored to investigate the effect of upregulated expression of GIMAPs on the growth and proliferation of *N. caninum* (a close relative of *T. gondii*) in rat and bovine cells.

## 2 Materials and methods

### 2.1 Parasites

*N. caninum* Nc-1 GFP-expressing strain (a generous gift from Dr. Jeroen Saeij, University of California, Davis) was propagated by culturing in human foreskin fibroblasts (HFF). Prior to infection with *N. caninum*, HFF monolayers were grown to confluence in Iscove’s modified Dulbecco’s medium supplemented with 10% (vol/vol) heat-inactivated fetal bovine serum, 1% (vol/vol) GlutaMAX, and 1% (vol/vol) penicillin-streptomycin-amphotericin B (Fungizone) (Life Technologies) at 37 °C with 5% CO_2_. To extract *N. caninum* tachyzoites, infected HFF were detached and resuspended in culture medium. Tachyzoites were extruded by passing the cell suspension twice through a 25-gauge needle. Subsequently, tachyzoites were separated from the cell debris by passing through a 3-μm filter, followed by washing thrice in sterile phosphate-buffered saline (PBS), and enumeration using a hemocytometer.

### 2.2 Rat primary cells and *in-vivo* infection assays

The University of Illinois Urbana–Champaign Institutional Animal Care and Use Committee-approved protocol number 22212 was used for the care and use of rats in this study. Six-week-old female Lewis (LEW) and Brown Norway (BN) rats were purchased from Charles River, USA. For extraction of rat primary peritoneal cells, following acclimatization, three rats from each strain were sacrificed using CO_2_ asphyxiation by placing the animals in a chamber and introducing 100% compressed CO_2_ at a displacement rate of 50% of the chamber volume per minute. Once respiration ceased, death of the animal was confirmed by cervical dislocation. Immediately peritoneal cells were harvested by peritoneal lavage with 20 ml of IMDM medium. Peritoneal cells were washed three times in IMDM medium followed by enumeration. The peritoneal cells were seeded in 12-well plates at a density of 1.6 × 10^6^/well in supplemented IMDM medium (with 10% fetal bovine serum, 1 × nonessential amino acids [Sigma], 2.05 mM L-glutamine, and 1% penicillin-streptomycin [Life Technologies]) and infected with freshly isolated *N. caninum* tachyzoites at a Multiplicity of Infection (MOI) ratio (Parasite:cells) of 1:10. Uninfected cells were maintained as controls. All infection assays were performed in triplicate. The cells were incubated at 37 °C with 5% CO_2._ At 24 and 48h post-infection (PI), one set of cultures was used for total RNA extraction with the TRIzol Reagent (Invitrogen) and quantified using a NanoDrop ND-1000 spectrophotometer (Thermo Scientific). For each RNA sample, 1 μg was treated with DNase I (Invitrogen) followed by cDNA synthesis using the Superscript Reverse Transcriptase III kit (Invitrogen). From other sets of cultures, at 0, 72, and 120h of culture post-infection, genomic DNA was extracted using the PureLink genomic DNA kit (Invitrogen) and quantified. The DNA concentrations in the various samples were diluted uniformly using molecular-grade sterile water and used as a template for quantification of *N. caninum* DNA in the cultures, while the cDNA was used for the quantification of the glyceraldehyde 3-phosphate dehydrogenase (GAPDH) and GIMAP 4, 5, and 6 transcripts in the peritoneal cells using quantitative real-time PCR assays.

For *in-vivo* infection assays, groups of LEW and BN rats (*n* = 5) were used. In infected groups, each rat was intraperitoneally inoculated with 5 × 10^6^ freshly isolated *N. caninum* tachyzoites suspended in 0.5 ml PBS, while in the control groups each rat was inoculated with 0.5 ml of sterile PBS. One set of rats were sacrificed at 72h PI, and peritoneal cells isolated immediately by peritoneal lavage. Rats for chronic infection was sacrificed at 8 weeks post-infection, and peritoneal cells were collected. Additionally, from these rats, organ (liver, spleen, uterus, muscle, and brain) samples were collected and submerged in PBS for genomic DNA isolation. For both the 72h and 8-week PI rat sets, 1.6 × 10^6^ cells per sample of the purified peritoneal cells were used for the extraction of total RNA and genomic DNA. For the organ samples, 10 mg of tissue was used for genomic DNA extraction. The DNA concentrations in the various samples were diluted uniformly using molecular-grade sterile. cDNA was synthesized from the RNA samples as described above and used for the quantification of GAPDH and GIMP transcripts, while the genomic DNA was used for quantification of *N. caninum* load.

### 2.3 Real-time PCR quantification of GIMAP transcripts and *N. caninum* load in rat cells and tissues

For quantification of GIMAP transcripts, the primer pairs used were RT-GIMAP 4-F and RT-GIMAP 4-R for GIMAP 4, RT-GIMAP 5-F and RT-GIMAP 5-R for GIMAP 5, RT-GIMAP 6-F and RT-GIMAP 6-R for GIMAP 6, and Rat-GAPDH-mRNA-F and Rat-GAPDH-mRNA-R for rat GAPDH (**Table 1**). Those primer pairs amplified cDNA fragments of 104 bp, 130 bp, 87 bp, and 170 bp for GIMPAP 4, GIMAP 5, GIMAP 6, and GAPDH transcripts, respectively. For *N. caninum* quantification, the primer pairs used were NC-5 F and NC-5 R, targeting a 150 bp *N. caninum* NC-5 gene fragment (GenBank Accession number: FJ464412); Rat-GAPDH-F and Rat-GAPDH-R targeting a 284 bp GAPDH gene fragment (**Table 1**). Conventional PCR products for each transcript from total cDNA or genomic DNA were fractioned on agarose gel, and the DNA bands were extracted using a QIAquick gel extraction kit (Qiagen). The concentration of the purified DNA fragments was determined, and 10-fold serial dilutions were generated for use as standards for creating standard curves for quantitative real-time PCR. Each real-time PCR mixture contained 1 μl of cDNA or genomic DNA template, 1 μl of primer mix (500 nM each primer), and 10 μl of PowerUp SYBR Green Master Mix (Applied Biosystems), with the final volume made up to 20 μl with sterile nuclease-free water. The cycling conditions included an initial denaturation for 10 min at 95 °C, 45 cycles at 98 °C for 15 s and 60 °C for 1 min, and a final melting curve step. Cycling was performed using a QuantStudio 3 Real-time PCR system (Applied Biosystems). The system software was used to generate standard curves for deriving transcript concentrations. The relative concentrations of the target amplicons were normalized using GAPDH amplicon concentrations.

**Table 1:** Primers used in this study.

Primer name	Primer sequence (5′-3′)
Rat-GAPDH-mRNA-F	GGATACTGAGAGCAAGAGAGA
Rat-GAPDH-mRNA-R	GGGTGCAGCGAACTTTAT
RT-GIMAP 4-F	CACTCGCTGTGTTGCTCTGA
RT-GIMAP 4-R	GAAGTTTCCGTGTGGCCTTG
RT-GIMAP 5-F	GCTTCCTAGTGGTGGACACG
RT-GIMAP 5-R	AGTTGGGTCACCAGCAACAA
RT-GIMAP 6-F	CAAAATCAGCGCTCGACCAG
RT-GIMAP 6-R	GGGGTGTCGATCACCTCAAG
NC-5-F	TATAGTGTGTGAACGGGTGAAC
NC-5-R	ACAGAACACTGAACTCTCGATAAG
Rat-GAPDH-F	TTCCCTGAGTCCTATCCTGGGAA
Rat-GAPDH-R	TTATAGGAACTGGATGGTGGGGG
BOV-RT-GIMAP-4-F	GAGAGCTGATTCGTGAGTTC
BOV-RT-GIMAP-4-R	CTCCTCGGTCTTCTGATACA
BOV-RT-GIMAP-5-F	CCAGGATCAAGAGGTGTATG
BOV-RT-GIMAP-5-R	GTGAAGAGGATGACCATGTAT
BOV-RT-GIMAP-6-F	ACACAGAGAGTTCAGGAGAG
BOV-RT-GIMAP-6-R	GAGAAGGATCCTGGGATG
BOV-GAPDH-F	GAGATCAAGAAGGTGGTGAAG
BOV-GAPDH-R	TGACAAAGTGGTCGTTGAG
BOV-ORF-pLVX-G-4-F	CCCTCGTAAAGAATTCATGGCAGCCCAGTACCTCAG
BOV-ORF-pLVX-G-4-R	GAGGTGGTCTGGATCCTTAATCCCTAAACAGAGGGA
BOV-ORF-pLVX-G-5-F	CCCTCGTAAAGAATTCATGGAAGGGCTTCAGAGGAG
BOV-ORF-pLVX-G-5-R	GAGGTGGTCTGGATCCTCAGGTCTTGCTTTTGCTTT
BOV-ORF-pLVX-G-6-F	CCCTCGTAAAGAATTCATGTCCTCTATGTTTGTGTA
BOV-ORF-pLVX-G6-R	GAGGTGGTCTGGATCCTCAGATGGGCGTGCTGGGTG
pLVX-F	ATGTAAACCAGGGCGCCTAT
pLVX-R	ACCCGTCTTTGGATTAGGCA

### 2.4 Analysis of *N. caninum* effect on endogenous GIMAPs in a bovine macrophage cell line

A bovine macrophage (BoMAC) cell line (a generous gift from Prof. Matthias Schweizer, Institute of Virology and Immunology, Switzerland) was cultured in IMDM medium supplemented with 1× MEM nonessential amino acids (Sigma), 1× OPI Media Supplement-Hybri-Max (Sigma), 1× 2-mercaptoethanol (Gibco), 1.5 mg/ml sodium bicarbonate, 10% (v/v) heat-inactivated fetal bovine serum, 100 U/ml penicillin, and 100 µg/ml streptomycin. One set of cells was maintained uninfected, while another set was infected with freshly extracted tachyzoites of *N. caninum* at an MOI of 1:10. At 36h PI, the cells were harvested and total RNA extracted using the TRIzol reagent. One microgram of the total RNA was treated with DNase-I and used to synthesize cDNA with the SuperScript II Reverse Transcriptase kit. Real-time PCR was used to quantify GIMAPs and GAPDH transcripts in 1 μl of cDNA using the following primer pairs: BOV-RT-GIMAP-4-F and BOV-RT-GIMAP-4-R for GIMAP 4 (GenBank accession number BC114879); BOV-RT-GIMAP-5-F and BOV-RT-GIMAP-5-R for GIMAP 5 (GenBank accession number BC103445); BOV-RT-GIMAP-6-F and BOV-RT-GIMAP-6-R for GIMAP 6 (GenBank accession number BC142429); and BOV-GAPDH-F and BOV-GAPDH-R for GAPDH (GenBank accession number BC102589) (**Table 1**). The relative concentrations of the target transcripts were normalized using GAPDH transcript concentrations.

### 2.5 Generation of lentiviral particles for inducible expression of bovine GIMAP genes

To amplify full coding sequences for bovine GIMAP genes, the following primer sets were used: Bov-ORF-pLVX-G-4-F and Bov-ORF-pLVX-G-4-R for GIMAP 4 (GenBank accession number: BC114879); Bov-ORF-pLVX-G-5-F and Bov-ORF-pLVX-G-5-R for GIMAP 5 (GenBank accession number: BC103445); and Bov-ORF-pLVX-G-6-F and Bov-ORF-pLVX-G-6-R for GIMAP 6 (GenBank accession number BC142429) (**Table 1**). The respective coding sequences were amplified from BoMAC cDNA using the CloneAmp HiFi PCR Premix kit (Clontech, Takara Bio. California, USA), and the amplicons were cloned into the pLVX-TetOne-Puro expression vector using the In-Fusion HD Cloning System (Clontech, Takara Bio. California, USA) following the manufacturer’s instructions. The recombinant pLVX-TetOne-Puro expression vectors carrying the coding sequences or without gene insert were transformed in Stellar competent *E. coli* (Clontech, Takara Bio. California, USA) and purified using the NucleoBond Xtra Midi Plus (Clontech, Takara Bio. California, USA). Gene insert identity was confirmed by sequencing, following which the purified pLVX-TetOne-Puro expression plasmids were used in combination with the Lenti-X Packaging Single Shots (Clontech, Takara Bio. California, USA) to generate lentivirus particles in Lenti-X 293T cells (Clontech, Takara Bio. California, USA). Briefly, Lenti-X 293T cells were grown to 80% confluence in supplemented DMEM medium (4.5 mg/ml glucose, 4 mM L-glutamine, 1 mM sodium pyruvate, 1.5 mg/ml sodium bicarbonate, 10% heat-inactivated fetal bovine serum, 100 U/ml penicillin, and 100 µg/ml streptomycin). Transfections were performed using 7.0 µg of recombinant pLVX-TetOne-Puro expression plasmid in 600 µl of sterile nuclease-free water mixed with a Lenti-X Packaging Single Shot and incubated for 10 min at room temperature. The mixture was added dropwise to Lenti-X 293T cells cultured in a 10 cm diameter petri dish and mixed by gentle rocking. The cells were incubated at 37°C with 5% CO_2,_ and the medium was changed after 8h and incubated for a further 48h. The culture medium was collected, and the lentiviral particles were concentrated using the Lenti-X Concentrator reagent (Clontech, Takara Bio. California, USA), following which the particle titer was estimated using the Lenti-X GoStix reagent. The viral particles were aliquoted and stored at −80°C until use.

### 2.6 Transduction of BoMAC cells with lentiviral particles

Lentiviral particles expressing the GIMAP transgenes or the empty pLVX-TetOne-Puro expression vector (control) were used to transduce BoMAC cells. Briefly, BoMAC cells were cultured to 70% confluence in 10 cm diameter petri dishes with IMDM medium supplemented with 1× MEM nonessential amino acids (Sigma), 1× OPI media supplement-Hybri-Max (Sigma), 1× 2-mercaptoethanol (Gibco), 1.5 mg/ml sodium bicarbonate, 10% (v/v) heat-inactivated fetal bovine serum (GIBCO, certified tetracycline-free), 100 U/ml penicillin, and 100 µg/ml streptomycin, following which polybrene solution (4 μg/ml final concentration) was added and mixed by gentle rocking. Next, 200 μl of lentiviral aliquot (about 10^5^ IFU) was added to the cells dropwise and mixed by gentle rocking, and the cells were cultured overnight. Puromycin (12 µg/ml) (Clontech, Takara Bio. California, USA) selection was performed for 3 weeks, and the ensuing resistant transformants were cloned by limiting dilution. Selected clones were cultured in the absence or presence of 1 μg/ml doxycycline. Total RNA was extracted from the cells, and 1 μg was treated with DNaseI, and cDNA was synthesized using the SuperScript II Reverse Transcriptase kit. Real-time PCR was used to quantify GIMAPs and GAPDH transcript expression using the following primer pairs: BOV-RT-GIMAP-4-F and BOV-RT-GIMAP-4-R for GIMAP 4, BOV-RT-GIMAP-5-F and BOV-RT-GIMAP-5-R for GIMAP 5, BOV-RT-GIMAP-6-F and BOV-RT-GIMAP-6-R for GIMAP 6, and BOV-GAPDH-F and BOV-GAPDH-R for GAPDH (**Table 1**). Real-time PCR mixtures contained 1 μl of cDNA template, 1 μl of primer mix (500 nM of each primer), and 10 μl of PowerUp SYBR Green Master Mix, with the final volume made up to 20 μl with sterile nuclease-free water. The cycling conditions included an initial denaturation for 10 min at 95°C, 45 cycles at 98°C for 15 s and 60°C for 1 min, and a final melting curve step. Cycling was performed using the QuantStudio 3 real-time PCR system. The system software was used to generate standard curves for deriving transcript concentrations. The relative concentrations of the target transcripts were normalized using GAPDH transcript concentrations.

### 2.7 Analysis of GIMAPs’ effect on *N. caninum* growth in rat and bovine macrophage cell lines

In our previous work, we had generated a rat macrophage cell line (NR8383) for inducible overexpression of individual rat GIMAP 4, 5, and 6 transgenes or the pLVX-TetOne-Puro empty vector (Kim et al., 2018). The wild-type NR8383 cell line is permissive for *N. caninum* infection and intracellular proliferation. Thus, to determine the effect of inducible expression of GIMAPs on *N. caninum* growth and proliferation in the NR8383 cells, the transgenic NR8383 cells were used. On the other hand, the BoMAC cell line that is permissive for *N. caninum* infection and proliferation was engineered for inducible expression of bovine GIMAP 4, 5, and 6 in order to determine the effect of overexpression of the transgenes on *N. caninum* intracellular growth. NR8383 cells were cultured in IMDM medium supplemented with 1× MEM nonessential amino acids (Sigma), 1× OPI media supplement-Hybri-Max (Sigma), 1.5 mg/ml sodium bicarbonate, 10% (v/v) heat-inactivated fetal bovine serum (certified tetracycline free), 100 U/ml penicillin, and 100 µg/ml streptomycin. BoMAC cells were cultured in IMDM supplemented with 1× MEM Nonessential amino acids (Sigma), 1× OPI Media Supplement-Hybri-Max (Sigma), 1× 2-Mercaptoethanol (Gibco), 1.5 mg/ml sodium bicarbonate, 10% (v/v) heat-inactivated fetal bovine serum (GIBCO - certified tetracycline-free), 100 U/ml penicillin, and 100 µg/ml streptomycin. Cells were grown to confluence in 12-well plates, and the medium was replaced with fresh medium containing 1 μg/ml doxycycline and cultured for a further 24h, following which fresh medium with 1 μg/ml doxycycline was added and the cells inoculated with freshly extracted *N. caninum* tachyzoites at an MOI of 1:10. At 0h and 48h PI, genomic DNA was extracted from the whole culture of each well using the PureLink Genomic DNA Kits. The NC-5 gene and the rat GAPDH (GenBank accession number: BC029618) or bovine GAPDH (GenBank accession number: BC102589) gene fragments were targeted for quantification by real-time PCR. The relative amount of load of *N. caninum* was derived by dividing the concentration of the NC-5 gene by the concentration of the GAPDH gene from each DNA sample.

### 2.8 Immunofluorescence assays

Transgenic NR8383 cells induced to express GIMAP 4, 5, or 6 transgenes, and those carrying the empty expression vector (pLVX-TetOne-Puro) were grown in supplemented IMDM and infected with *N. caninum* NC-1 strain tachyzoites at an MOI of 1:10. About 48h PI, the cultures were fixed with 3% formaldehyde in PBS for 30 min at room temperature, following which the formaldehyde was rinsed off with PBS and the cells permeabilized with 0.2% Triton X-100 in PBS for 10 min at room temperature. After washing with PBS, the cells were incubated overnight at 4°C with primary antibodies in blocking buffer (0.1% Triton X-100, 3% bovine serum albumin, and 3% normal goat serum in PBS). The primary antibodies used were rabbit anti-LAMP1 (Santa Cruz, Biotechnology) and mouse anti-GFP monoclonal antibody (Abnova) diluted at a ratio of 1:500. Following three washes with blocking buffer, secondary antibodies [anti-rat-IgG-FITC (Sigma) and goat-anti-mouse IgG (H+L) Texas red dye conjugate (Sigma)] diluted in the blocking buffer at 1:500 were added to the cells and incubated at room temperature for 1h. The cells were then washed twice with PBS, air-dried, a drop of ProLong Gold antifade DAPI reagent (Invitrogen) added, and a cover slip placed on top followed by sealing with nail polish. Fluorescent images were captured using a Keyence BZ-X800 series fluorescence microscope system and analyzed using the BZ-X800 analyzer software.

### 2.9 LysoTracker assay for intracellular acidification

To determine the effect of upregulated expression of GIMAPs on induction of acidification of the intracellular parasite vacuoles, transgenic NR8383 cells induced to express GIMAP 4, 5, or 6 transgenes, and those carrying the empty expression vector (pLVX-TetOne-Puro) were grown in supplemented IMDM and infected with viable or heat-inactivated *N. caninum* NC-1 strain tachyzoites at an MOI of 1:10. At 48h PI, the medium in the culture was replaced with fresh medium containing 50 nM of LysoTracker Deep Red (Invitrogen) and 2 µg/ml of Hoechst 33258 (Invitrogen). Following 30 min of incubation, the cultures were analyzed by fluorescence microscopy as described above in section 2.8.

### 2.10 Statistical analyses

Statistical analyses were performed using two-tailed Student’s *t* test with GraphPad PRISM v8 software *P*-values of 0.05 or less were considered significant.

## 3 Results

### 3.1 LEW rat augments GIMAP 4, 5, and 6 expressions, and is refractory to *N. caninum* infection

**Figure 1:**
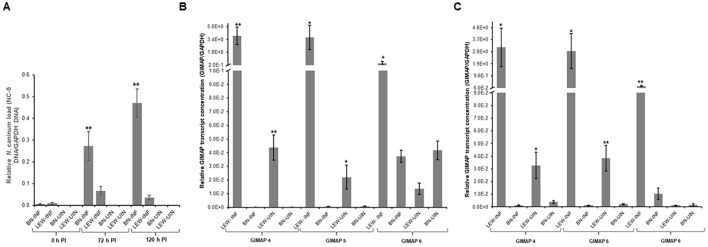
Real-time PCR quantification of the load of *N. caninum* and GIMAPs expression in primary rat peritoneal cells cultured *in vitro*. **(A)** Load of *N. caninum* NC-5 gene amplicon in LEW and BN rat peritoneal cells with or without *N. caninum* infection at 0h, 72h, and 120h post-infection. The relative concentration of NC-5 was derived by dividing its concentration with that of rat GAPDH in the same sample. **(B, C)** Relative expression of GIMAPs at **(B)** 24h, and **(C)** 48 h post-infection of LEW or BN rat peritoneal cells with *N. caninum*. The normalized concentration of GIMAP 4, 5, or 6 transcript was derived by dividing the concentration of the respective GIMAP transcript by the concentration of rat GAPDH transcript in the same sample. The data shown represent means of three independent experiments with standard error bars and levels of statistical significance difference relative to the LEW-INF group depicted by asterisks (**P < 0.001). LEW-INF (infected LEW rat cells); LEW-UIN (uninfected LEW rat cells); BN-INF (infected BN rat cells); BN-UIN (uninfected BN rat cells). (*P < 0.05).

We endeavored to compare the susceptibility of the BN and LEW rat to *N. caninum* infection and determine the effect on expression of GIMAPs in the rats’ peritoneal cells. When freshly extracted peritoneal cells from LEW and BN rats were cultured and infected with *N. caninum*, analysis of the parasite load depicted a time-dependent suppression of parasite proliferation in the LEW rat’s peritoneal cells, while the parasites progressively proliferated in the BN rat’s cells (**Figure 1**). Notably, at 72h post-infection (PI), the parasite load in the BN rat cells was about 5.5-fold higher than that in the LEW rat cells (**Figure 1A**). With an increase in time of culture, the parasite load in the LEW rat cells declined, while that in the BN rat cells increased, being about 23-fold higher than in the LEW rat cells (**Figure 1**). This indicated that the LEW rat peritoneal cells were refractory to *N. caninum* infection, while the BN rat cells were permissive, consistent with the earlier observed responses to *T. gondii* infection (Kim et al., 2018). Analysis of the expression of GIMAPs showed that, at 24h PI, the infected LEW rat peritoneal cells had approximately 340-fold, 320-fold, and 50-fold higher expression of GIMAP 4, 5, and 6, respectively, than in the BN rat cells (**Figure 1B**). A similar trend was evident at 48 h PI, with LEW rats having 280-fold, 230-fold, and 6-fold higher expression of GIMAP 4, 5, and 6, respectively, than in the BN rats (**Figure 1C**). While the uninfected LEW rats also had notably higher expression levels of GIMAP 4, 5, and 6 than the uninfected BN rats, the magnitude of difference was hundreds of fold lower than that in infected rats (**Figures 1B, C**). These findings indicated that *N. caninum* infection in LEW rat augments expression of GIMAPs more than in BN rats, which corresponds to the refractoriness of the LEW rat to the infection in comparison to the *N. caninum*-permissive BN rats.

**Figure 2:**
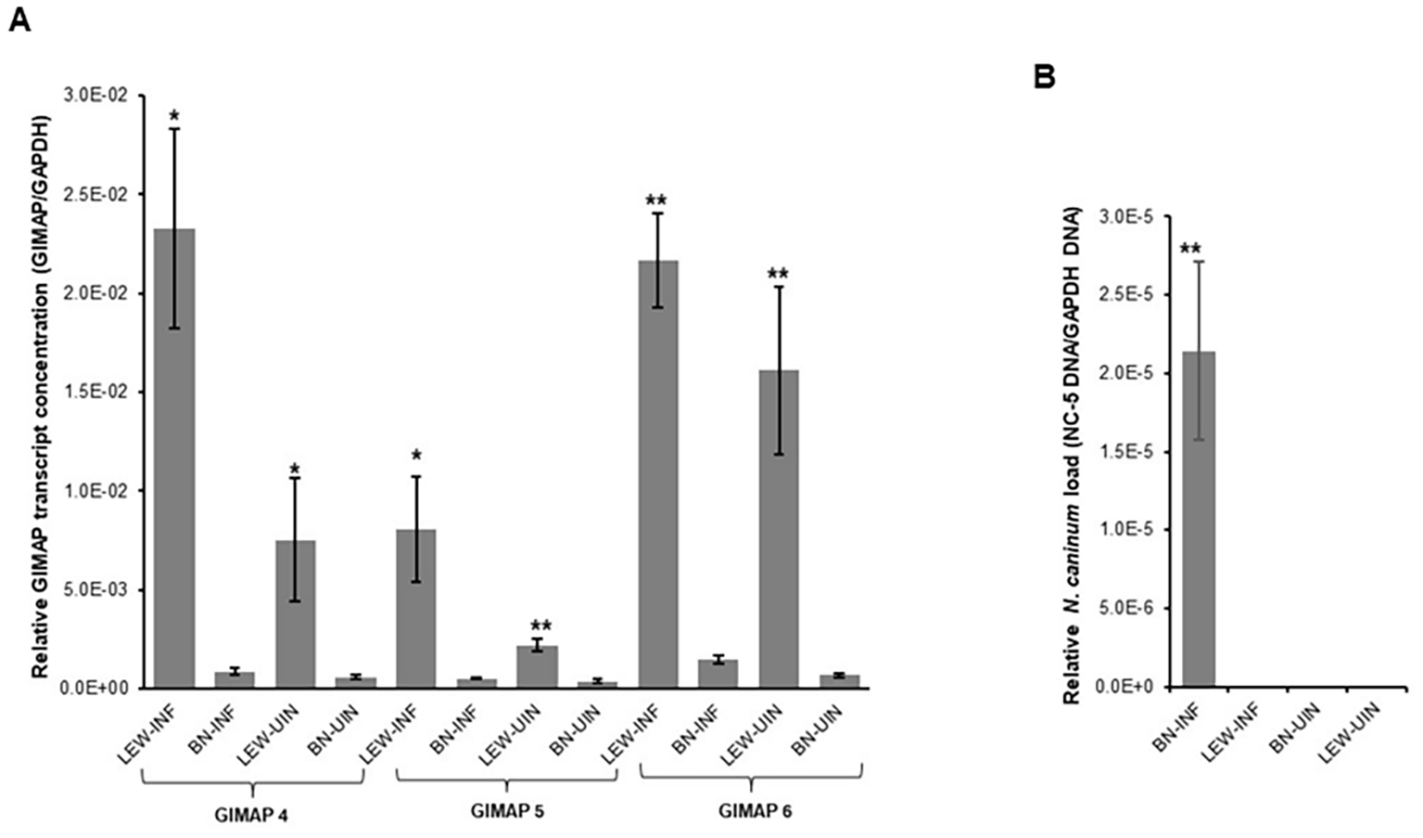
Real time PCR quantification of the *in vivo* expression of GIMAPs, and *N. caninum* load in LEW and BN rats. At 72h post-infection with *N. caninum* total RNA and genomic DNA were extracted from LEW and BN rats’ peritoneal cells. **(A)** GIMAP 4, 5, and 6 transcripts were quantified in the cDNA synthesized from the RNA. The relative concentration of each GIMAP transcript was normalized with rat GAPDH transcripts in the same sample. **(B)** The concentration of *N. caninum* NC-5 gene was quantified in genomic DNA and normalized with that of rat GAPDH gene in the same sample. The data shown represent means of three independent experiments with standard error bars and levels of statistical significance relative to the BN-INF group depicted by asterisks (*P < 0.05; **P < 0.001). LEW-INF (infected LEW rat cells); LEW-UIN (uninfected LEW rat cells); BN-INF (infected BN rat cells); BN-UIN (uninfected BN rat cells).

**Figure 3:**
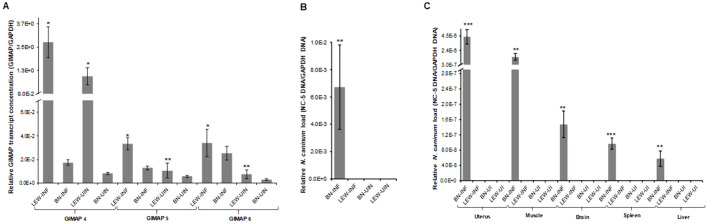
Real time PCR quantification of the *in vivo* expression of GIMAPs, and *N. caninum* load during chronic infection of LEW and BN rats. **(A)** At 8 weeks post-infection GIMAP 4, 5, and 6 transcripts were quantified by Real Time PCR using cDNA synthesized from RNA extracted from LEW and BN rat peritoneal cells. GIMAP transcripts were normalized with rat GAPDH transcripts in the same sample. **(B, C)** Genomic DNA extracted from the rats’ peritoneal cells **(B)** or organ tissue including uterus, muscle, brain, spleen, and liver **(C)** was used to quantify the concentration of *N. caninum* NC-5 and rat GAPDH genes. The relative concentration of NC-5 was normalized with that of rat GAPDH in the same sample. The data shown represent means of three independent experiments with standard error bars and levels of statistical significance difference relative to the BN-INF group depicted by asterisks (**P < 0.001). LEW-INF (infected LEW rat cells); LEW-UIN (uninfected LEW rat cells); BN-INF (infected BN rat cells); BN-UIN (uninfected BN rat cells). (*P < 0.05), (***P < 0.0001)..

Next, we assessed the *in vivo N. caninum* infection dynamics in LEW and BN rats with regard to parasite proliferation and GIMAP expression levels. Consistent with the *in-vitro* assays, LEW rats were refractory to *N. caninum* infection, while the BN rats were permissive, showing over 6,000-fold higher than baseline parasite load in peritoneal cells 72h following intraperitoneal inoculation of *N. caninum* tachyzoites (**Figure 2B**). Concomitantly, infected LEW rats augmented expression of GIMAP 4, 5, and 6 by over 225-fold, 75-fold, and 200-fold, respectively, higher than the infected BN rats (**Figure 2A**). Notably, while the uninfected LEW rats had significantly higher expression levels of GIMAP 4 and 5 than the uninfected BN rats, those levels were over 50-fold lower than in the infected LEW rats (**Figure 2A**). Further, we analyzed the outcome of chronic *N. caninum* infection in LEW and BN rats. At 8 weeks PI, the LEW rats’ peritoneal cells were found to maintain significantly higher expression levels of GIMAP 4, 5, and 6 than the infected BN rats (**Figure 3A**). However, the magnitude of difference for GIMAP 6 was notably narrower than that of GIMAP 4 and 5 (**Figure 3A**). Consistently, the parasites were undetectable in the peritoneal cells from LEW rats, while the BN rats’ peritoneal cells had about 6,000-fold higher parasite load than the baseline infection level (**Figure 3B**). Analysis of parasite load in the various organ tissues showed that, while *N. caninum* DNA was undetectable in the LEW rats, there were significant detectable levels of *N. caninum* DNA in the uterus, muscle, brain, spleen, and liver of BN rats 8 weeks PI (**Figure 3C**). Together, these findings confirmed that the LEW rat is highly resistant to *N. caninum* infection, and that the infection induces the upregulation of expression of GIMAP 4, 5, and 6 genes.

### 3.2 GIMAP upregulation in rat NR8383 cells blocks intracellular *N. caninum* proliferation

**Figure 4:**
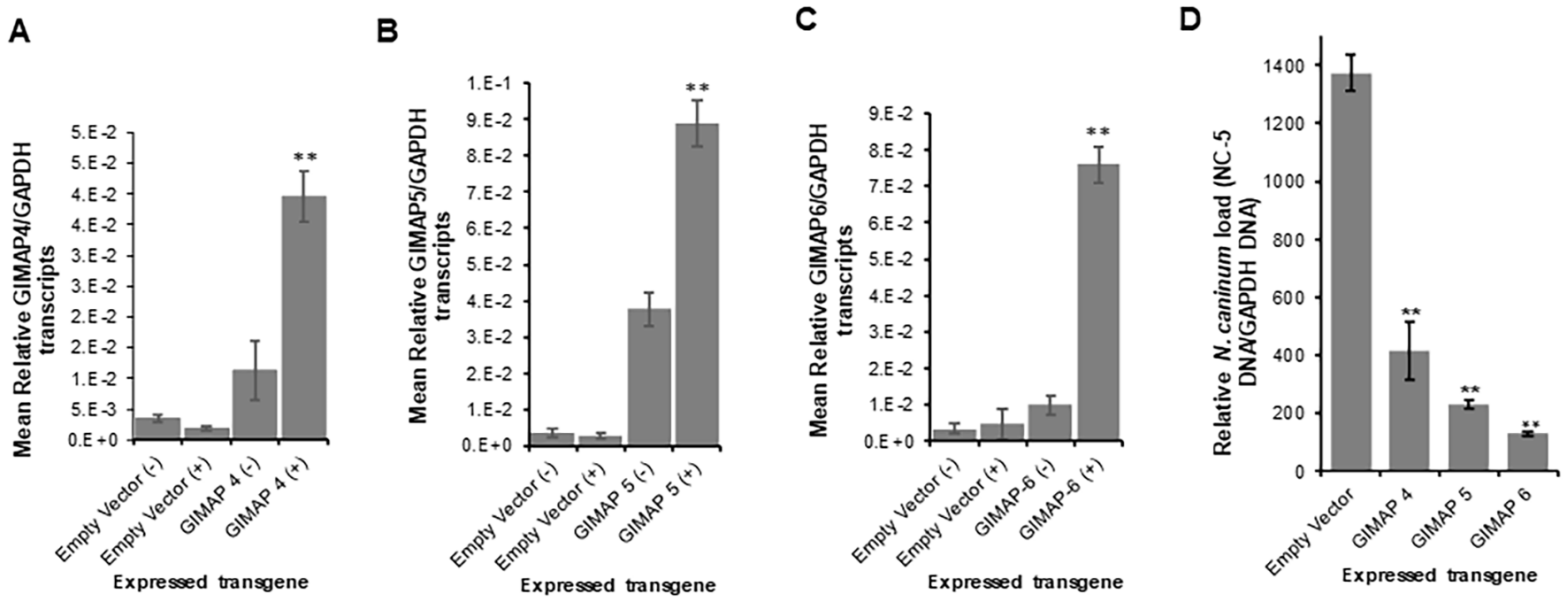
Quantification of the expression of GIMAP 4, 5, and 6, and *N. caninum* load in transgenic rat NR8383 macrophage cell line. cDNA was synthesized from RNA extracted from the cells induced or uninduced for expression of **(A)** GIMAP 4, **(B)** GIMAP 5, or **(C)** GIMAP 6 for 24h, and the expression of GIMAP 4, 5, and 6, and rat GAPDH transcripts quantified by Real-time PCR. GIMAP transcripts were normalized with rat GAPDH transcripts in the same sample. **(D)** After 24 h of induction of GIMAP expression, the cells were infected with fresh *N. caninum*, and 48 h post-infection total genomic DNA was extracted and the concentration of *N. caninum* NC-5 and rat GAPDH gene determined. The NC-5 concentration was normalized with the rat GAPDH in the same sample. The data shown represent means for three independent experiments with standard error bars and levels of statistical significance difference in parasite growth relative to that in the uninduced or Empty Vector cells depicted by asterisks (**P < 0.001).

We cultured the transgenic cells with or without doxycycline induction for 24h and found significant upregulation of GIMAP 4, 5, and 6 transcripts in the respective induced transgenic cells (**Figures 4A–C**). At 24h PI, the cells were infected with *N. caninum* tachyzoites, and at different time points of culture, we quantified the growth of the parasites in the cultures by real-time PCR, targeting the amplification of the *N. caninum* NC-5 gene. At 48h PI, we found that, while the parasites had proliferated in NR8383 cells with the empty expression vector, there were approximately 3.5-fold, 6.5-fold, and ninefold lower parasites in cell cultures overexpressing GIMAP 4, 5, and 6, respectively (**Figure 4D**). Notably, GIMAP 6 upregulation had the most restrictive effect on parasite growth, followed by GIMAP 5 and 4, in that order (**Figure 4D**). Together, these findings indicated that upregulation of the GIMAPs mediates restriction of intracellular *N. caninum* growth in rat macrophages consistent with the observations in LEW rats (that upregulate GIMAPs in response to infection) in comparison to the BN rats (that do not upregulate GIMAPs in response to infection).

### 3.3 Upregulation of GIMAPs induces convergence of lysosomes at the parasitophorous vacuole membrane and acidification of the vacuole

**Figure 5:**
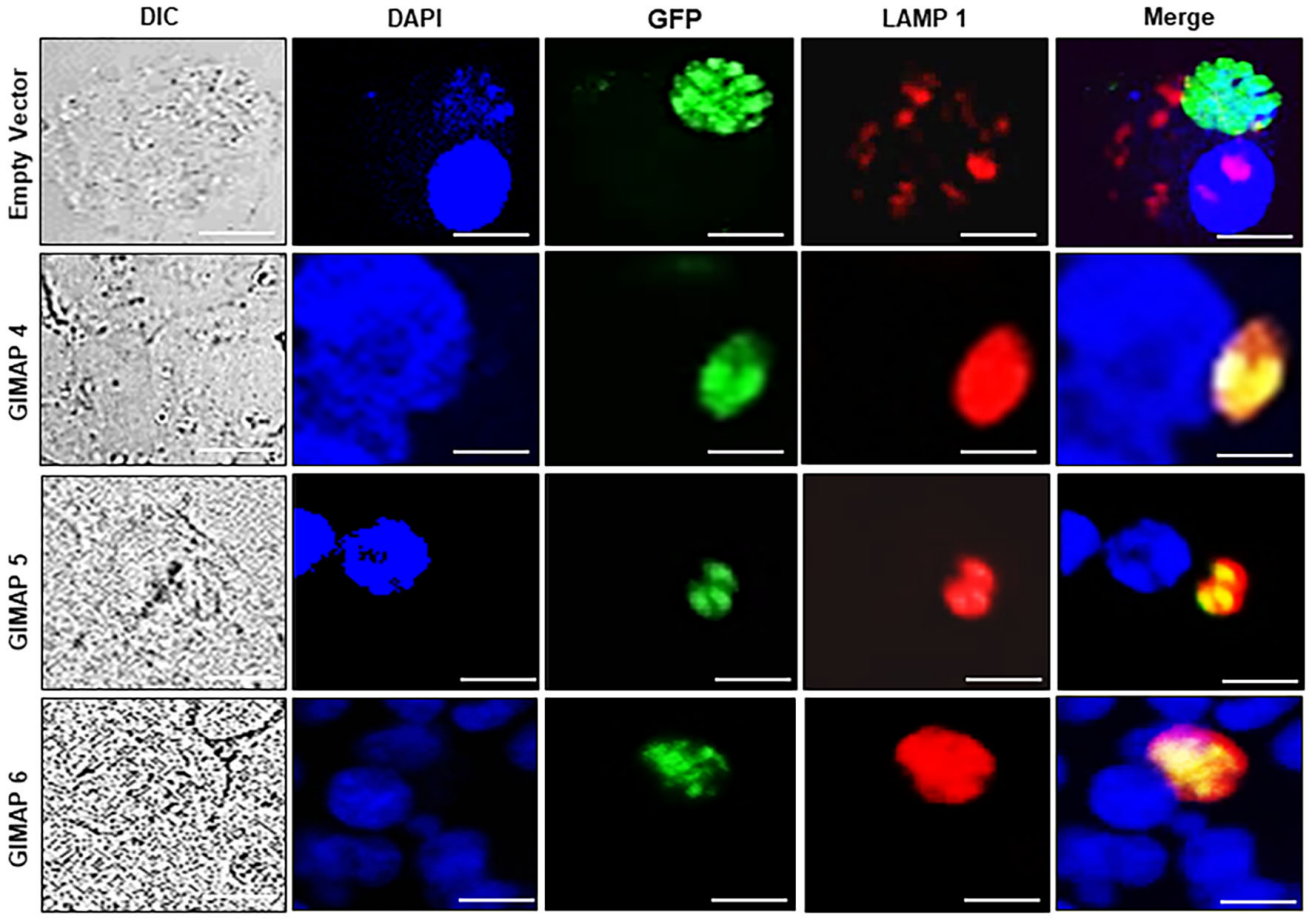
Immunofluorescence co-localization of the lysosome marker protein, LAMP1, with *N. caninum* tachyzoites in NR8383 cells. Transgenic NR8383 cells overexpressing GIMAP 4, 5, 6, or the empty pLVX expression vector (Empty Vector) were infected with *N. caninum* tachyzoites constitutively expressing green fluorescent protein (GFP) and cultured for 48 h. Immunofluorescence analysis was performed using a combination of DAPI nucleic acid stain (blue), anti-LAMP1 antibody (red), and anti-GFP antibody (green). In GIMAP 4, 5, and 6-expressing cells, LAMP1 densely co-localized with GFP (Merge panels), while in the cells expressing the empty vector, LAMP1 was sparsely distributed (Merge panels). Images of the cells were also captured by differential interference contrast (DIC). Imaging was done using a BZ-X800 series fluorescence microscope with a ×40 objective and analyzed with the BZ-X800 analyzer software. Scale bars, 20 µM.

**Figure 6:**
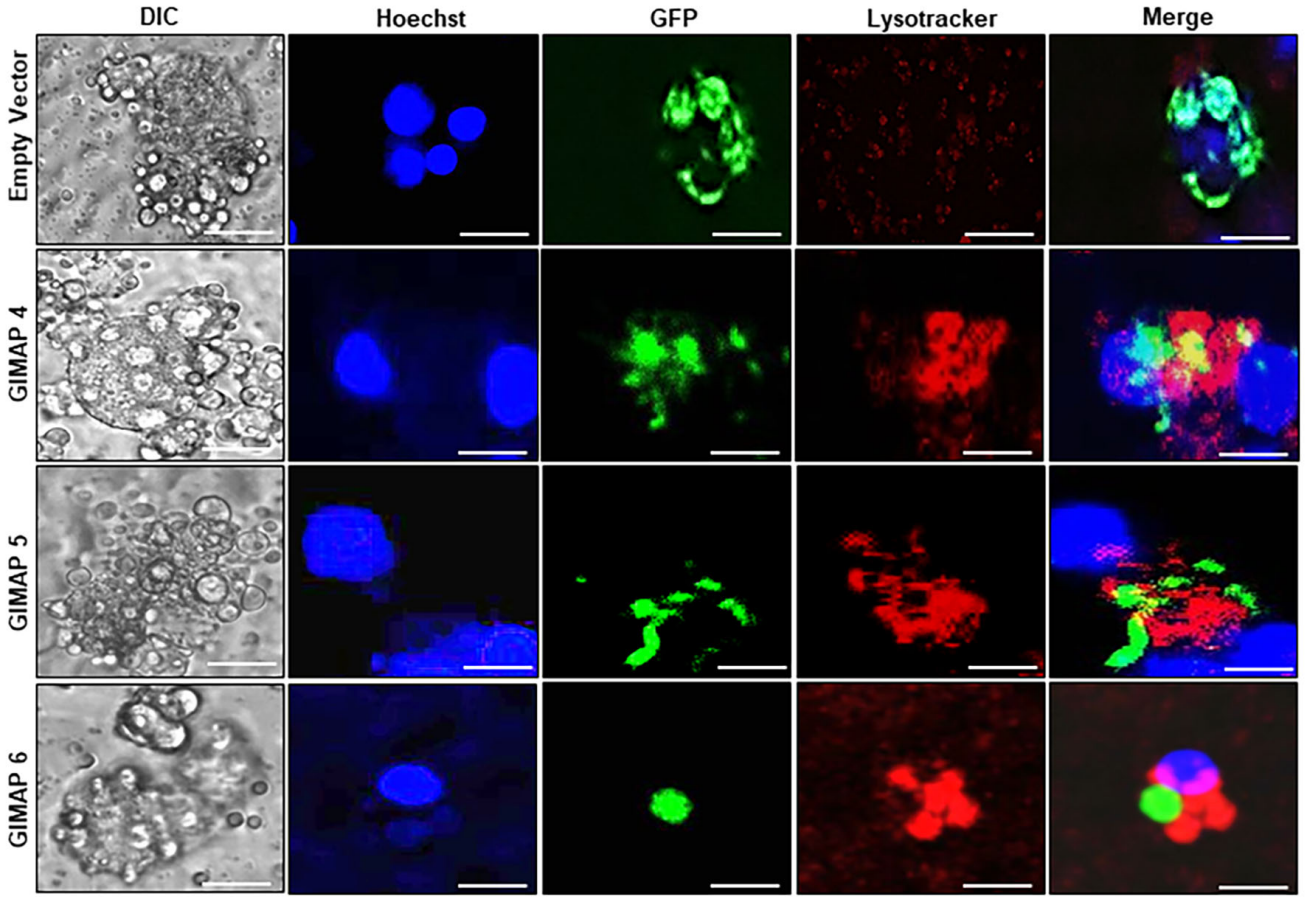
Analysis of the lysosomal acidification of the *N. caninum-*infected NR8383 macrophage cells. Transgenic NR8383 cells overexpressing GIMAP 4, 5, 6, or the empty pLVX expression vector (Empty Vector) were infected with *N. caninum* tachyzoites constitutively expressing green fluorescent protein (GFP) and cultured for 48 h. The cultures were treated with Hoechst stain (blue) and Lysotracker Deep Red (red) followed by fluorescence microscopy analysis. GIMAP 4, 5, and 6-expressing cells depicted prominent lysotracker staining overlapping the parasite green fluorescence, while cells expressing the empty vector showed faint, sparsely distributed lysotracker stain (Merge). Images of the cells were also captured by differential interference contrast (DIC). Imaging was done using a BZ-X800 series fluorescence microscope and analyzed with the BZ-X800 analyzer software. Scale bars, 20 µM.

We performed immunofluorescence assays to analyze the localization of LAMP 1 (a lysosome marker protein) in infected NR8383 cells induced for expressing the empty vector as a control, or GIMAP 4, 5, and 6. After 48h PI, the parasites had proliferated, but there were only sparsely distributed low-intensity signals for LAMP 1 in cells expressing the empty vector (**Figure 5**). Interestingly, in cells expressing GIMAP 4, 5, or 6, there was restricted growth of the parasites, but with dense co-localization of tachyzoite GFP with LAMP 1 (**Figure 5**), suggesting lysosomal fusion to the parasitophorous vacuole membrane (PVM). Further, to investigate whether the accumulation of lysosomes on the PVM led to the acidification of the PV, we used the LysoTracker Deep Red reagent that detects intracellular acidification. We found that in cells infected with viable *N. caninum* without induction of GIMAP overexpression, there was very faint sparsely distributed signal of the LysoTracker, but with evident proliferating *N. caninum* tachyzoite packs (**Figure 6**). In contrast, in cells expressing GIMAP 4, 5, or 6, there were dense signals of the LysoTracker co-localizing with the *N. caninum* tachyzoites (**Figure 6**). Evidently, there were fewer and seemingly disintegrated tachyzoites in these cells compared to the cells not overexpressing GIMAPs (**Figure 6**). Quantification of the lysotracker intensity within the parasitophorous vacuoles showed that GIMAP-overexpressing cells had significantly higher lysotracker intensity than the cells expressing the empty vector (**Supplementary Figure S1**). In cells infected with heat-inactivated *N. caninum* tachyzoites there were no intracellular parasites nor acidification observed (**Supplementary Figure S2**). Together, these findings indicated that induced upregulation of GIMAP expression leads to the accumulation of lysosomes at the PVM and acidification of the PV, resulting in blockage of parasite proliferation.

### 3.4 Rat GIMAPs have conserved orthologs in bovine

**Figure 7:**
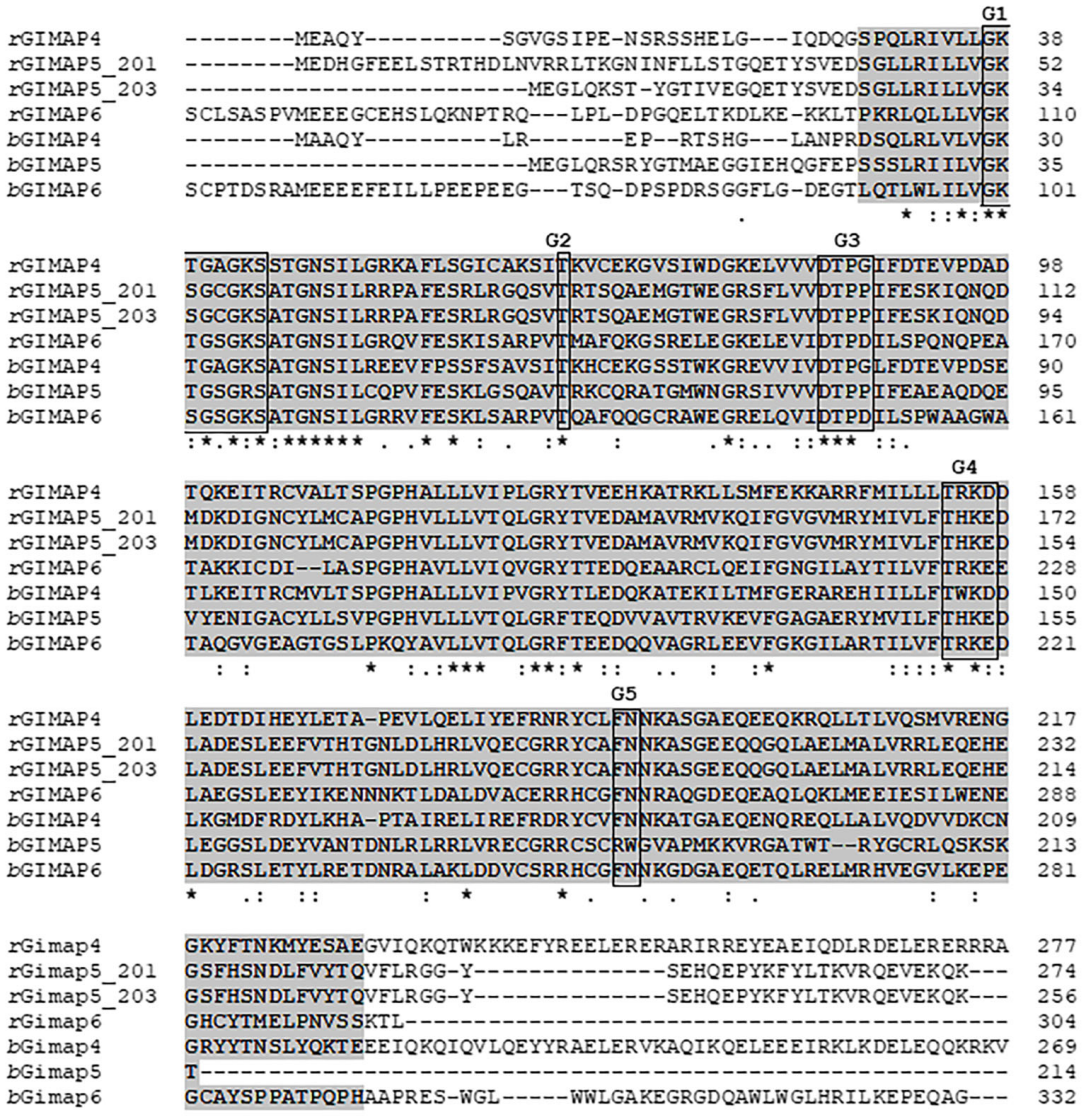
Alignment of the amino acid sequences for GIMAP 4, 5, and 6 encoded by LEW rat (*r*GIMAP) and bovine (*b*GIMAP) genes that were expressed as transgenes in NR8383 (rat macrophage) and BoMAC (bovine macrophage) cells, respectively. The amino acid sequences were aligned using the Clustal O (v1.2.4) program. The AIG1-type G domain common to all GIMAPs is highlighted in gray. The motifs G1, G2, G3, G4, and G5 are boxed. Asterisks indicate positions with a single, fully conserved residue.

Considering that *N. caninum* infection causes significant production losses in cattle, we cloned, sequenced, and analyzed the open reading frames of GIMAP 4, 5, and 6 from *Bos taurus* (bovine) cells. By sequence alignment we found that, just like the rat GIMAPs, bovine GIMAP 4, 5, and 6 have a conserved AIG1-type G domain containing a typical Walker A motif (G1), a Walker B motif (G3), as well as the G2, G4, and G5 motifs (**Figure 7**), characteristic of small GTPases (Krucken et al., 2004; Kim et al., 2018). By phylogenetic analysis we found that GIMAPs are evolutionarily conserved among mammalian species (**Supplementary Figure S3**).

### 3.5 Upregulation of GIMAP transgenes in bovine macrophages blocks *N. caninum* growth

**Figure 8:**
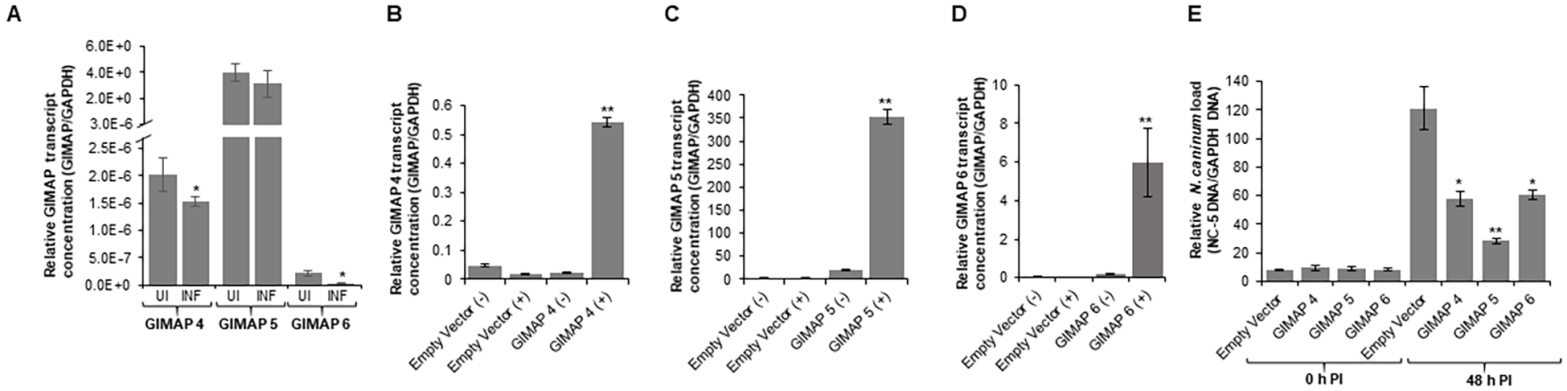
Quantification of the expression of GIMAP 4, 5, and 6, and load of *N. caninum* in wild-type and transgenic bovine macrophage (BoMAC) cells. **(A)** Wild-type BoMAC cells were cultured with or without *N. caninum* infection for 36 h, following which total RNA was extracted, cDNA synthesized and GIMAP 4, 5, and 6, and GAPDH transcripts were quantified by real-time PCR. GIMAP transcripts were normalized using bovine GAPDH transcripts in the same sample. UI (uninfected BoMAC cells); INF (infected BoMAC cells). **(B–D)** BoMAC cells engineered for expression of the pLVX expression vector (Empty Vector), bovine GIMAP 4, 5, or 6 were induced (+) or uninduced (−) for expression of the respective GIMAP transgene. After 24 h, total RNA was extracted and cDNA synthesized, following which GIMAP 4, 5, and 6, were quantified and normalized with bovine GAPDH transcripts in the same sample. **(E)** The cells were infected with *N. caninum*, and at 0 and 48 h post-infection, genomic DNA was extracted and used for quantification of *N. caninum* NC-5 and bovine GAPDH genes. NC-5 concentration was normalized with that of bovine GAPDH gene in the same sample. The data shown represent means for three independent experiments with standard error bars and levels of statistical significance difference in GIMAP expression relative to that in the uninfected or uninduced cells depicted by asterisks (*P < 0.05; **P < 0.001).

We determined the BoMAC cell line’s permissiveness to *N. caninum* infection and found that the parasites readily infected and proliferated in the wild-type BoMAC cells. Analysis of the expression of endogenous GIMAP 4, 5, and 6 in the cells depicted that *N. caninum* infection significantly downregulated the expression of GIMAP 4, 5, and 6 to levels lower than the baseline expression observed in uninfected cells (**Figure 8A**). To determine the effect of upregulation of bovine GIMAPs on *N. caninum* growth in BoMAC cells, we engineered BoMAC cells for inducible over-expression of bovine GIMAP 4, 5, or 6, as well as the empty expression vector. By quantitative Real-time PCR, we analyzed selected clones of the transgenic BoMAC cells and identified those that were doxycycline-inducible for overexpression of the GIMAP transgenes (**Figures 8B–D**). Using those selected clones expressing the GIMAPs, we infected the cells with tachyzoites of *N. caninum* with or without doxycycline induction and quantified the growth of the parasites in the cultures by real-time PCR, targeting the amplification of the *N.* caninum NC-5 gene. We found that, while the parasites proliferated in BoMAC cells expressing the empty vector, there were significant (*P* < 0.05) reductions in the growth of the parasites in cells overexpressing GIMAP 4, 5, or 6, with GIMAP 5 showing the most potent effect (**Figure 8E**). Together, these findings indicate that while *N. caninum* infection downregulates expression of GIMAPs in bovine macrophages, induced upregulated overexpression of GIMAP 4, 5, or 6 blocks *N. caninum* growth in bovine macrophages.

## 4 Discussion

The elucidation of *Neospora*-specific mechanisms that resistant mammalian hosts employ to orchestrate defenses against the parasite could unveil novel strategies for developing effective control approaches against neosporosis. Previously, we have shown that the LEW rat resists intracellular *T. gondii* growth by augmenting the expression of GIMAP 4, 5, and 6 in response to infection, that in-turn orchestrate the resistance phenotype (Kim et al., 2018). Herein, we investigated the effect of upregulated expression of GIMAPs on the growth and proliferation of *N. caninum* (an evolutionarily close relative of *T. gondii*) in rat and bovine cells.

Mammalian GIMAPs are small proteins, ranging in size from 34 kDa to 38 kDa (Filén and Lahesmaa, 2010), that are divergent from the larger IFN-γ-inducible GTPases (47–65 kDa), including immunity-related GTPases (IRGs) and guanylate-binding proteins (GBPs) that limit *T. gondii* infection in mice and humans, respectively (Boehm et al., 1998; Kim et al., 2012). In mammals, GIMAP genes are clustered, consistent with tandem gene duplications and tight co-regulation of gene expression (Krucken et al., 2004; Nitta and Takahama, 2007; Cury et al., 2024). We cloned, sequenced, and analyzed bovine GIMAP 4, 5 and 6 and found that, just like those in the rat, bovine GIMAP 4, 5, and 6 belong to the mammalian AIG family of GTPases that possess an AIG1 domain consisting of G1 through G5 boxes. The G1 box is the GTP-binding motif A (P-loop) that is important for hydrolysis of GTP (Schwefel et al., 2010; Tornabene et al., 2020). Between boxes G3 and G4, there is a unique conserved motif called consensus box CB, while the IAN (immune-associated nucleotide-binding protein) consensus sequence partially overlaps the G5 region (Reuber and Ausubel, 1996; Nitta and Takahama, 2007; Filén and Lahesmaa, 2010; Lu et al., 2020). In addition, all GIMAPs contain a helical segment that folds back on to the AIG1 domain and could be important in mediating protein-protein interactions (Limoges et al., 2021). The functional activity of GIMAPs is associated with the formation of dimers that typically dissociate when bound GTP is hydrolyzed by the G1 box motif (Tornabene et al., 2020). These notable structural features of AIG proteins can facilitate their oligomerization to the PVM that protects intracellular apicomplexan protozoan parasites, including *Toxoplasma* and *Neospora* (Filén and Lahesmaa, 2010; Howard et al., 2011).

We investigated the phenotypic responses of the LEW and BN rats to *N. caninum* infection. Similar to our earlier observed responses of the rats to *T. gondii* infection (Kim et al., 2018), we found that the LEW rat is refractory to *N. caninum* infection, while the BN rat is susceptible and develops a chronic infection. Consistently, we observed that, unlike the BN rat, the LEW rat augments the expression of GIMAP 4, 5, and 6 in response to *N. caninum* infection. The upregulation of GIMAPs is evident very early (24–48h) post-infection and is sustained over a period of several weeks, suggesting that the GIMAPs are involved in very early host innate mechanisms responsible for orchestrating the refractoriness of the LEW rat to *N. caninum* infection. Similarly, in snails, it has been shown that bacterial PAMPs upregulate the transcription of GIMAPs (Zhang et al., 2016). Corroboratively, knockdown of GIMAP 5 and 6 in human epithelial cells has been demonstrated to dramatically increase the infection rate of cells with herpes simplex virus 1 (HSV-1), with increased virus replication and viral particle production (Cury et al., 2024).

Therefore, to determine the roles of GIMAP 4, 5, and 6 in resistance to *N. caninum* infection in rats, we engineered transgenes of GIMAP 4, 5, and 6 for inducible overexpression in a *N. caninum*-susceptible NR8383 rat macrophage cell line. Overexpression of the transgenes induced accumulation of LAMP 1 (a lysosome marker protein) on the PVM resulting in parasite vacuole acidification, with concomitant restriction of *N. caninum* proliferation in the infected cells. Studies with *T. gondii* have demonstrated that lysosomal fusion to the otherwise non-fusogenic PVM leads to destruction of the intra-vacuole parasites (Andrade et al., 2006). Consistently, we have previously demonstrated the co-translocation of GIMAPs and lysosomes to the PVM in *T. gondii*-infected rat macrophages (Kim et al., 2018), while others have reported an IRG (IRGM1) that binds to intracellular *Mycobacterium bovis* phagosome membrane in association with SNARE protein complexes resulting in rapid fusion of lysosomes to the phagosome and destruction of the pathogen (Tiwari et al., 2009). GIMAPs have also been implicated in phagolysosomal processing in the coral *Acropora millepora* (Weiss et al., 2013).

Because *N. caninum* is known to inflict severe economic losses in cattle production through abortions and neonatal mortality, we endeavored to determine the effect of upregulation of GIMAPs on *N. caninum* survival and replication in a permissible bovine macrophage cell line (BoMAC). Interestingly, infection of the wild-type BoMAC cells with *N. caninum* significantly downregulated the transcription of GIMAP 4, 5, and 6 below the baseline levels, and the parasites proliferated progressively. Similarly, it has been shown that GIMAP 6 expression in cattle is downregulated in response to infection with *Mycobacterium avium* subspecies *paratuberculosis* (Thirunavukkarasu et al., 2014). While the exact mode of GIMAP activation by *N. caninum* in murine models, or downregulation in bovine, requires further investigation, it can be postulated that interaction of parasite molecules with the host’s unique transcriptional promoter elements could be contributing to the observed disparities in GIMAP expression among species and strains. Work to investigate this phenomenon could unveil possibilities for using GIMAP transcription activators to control infection. In this regard, we engineered BoMAC cells for inducible overexpression of individual bovine GIMAP 4, 5, or 6. Intriguingly, overexpression of GIMAP 4, 5, or 6 transgenes in BoMAC cells significantly blocked the proliferation of *N. caninum* tachyzoites, with GIMAP 5, showing the most potent effect. These observations were consistent with the anti-*Neospora* effect of upregulated expression of GIMAPs in rat cells. Collectively, our findings and those reported by others demonstrate that upregulation of expression of GIMAPs in cells of various species (rat, cattle, human, snails) confers innate resistance to intracellular invading pathogens (Tiwari et al., 2009; Kim et al., 2018; Lu et al., 2020; Cury et al., 2024).

## Data Availability

The datasets presented in this study can be found in online repositories. The names of the repository/repositories and accession number(s) can be found in the article/**Supplementary Material**.
