# Supplementary figures and images for: Upregulated small GTPase immunity-associated proteins confer resistance to Neospora caninum in rat and bovine cells
Source: Front Cell Infect Microbiol. 2025 Oct 14;15:1674380. doi: 10.3389/fcimb.2025.1674380 (PMC12558955; doi:10.3389/fcimb.2025.1674380)

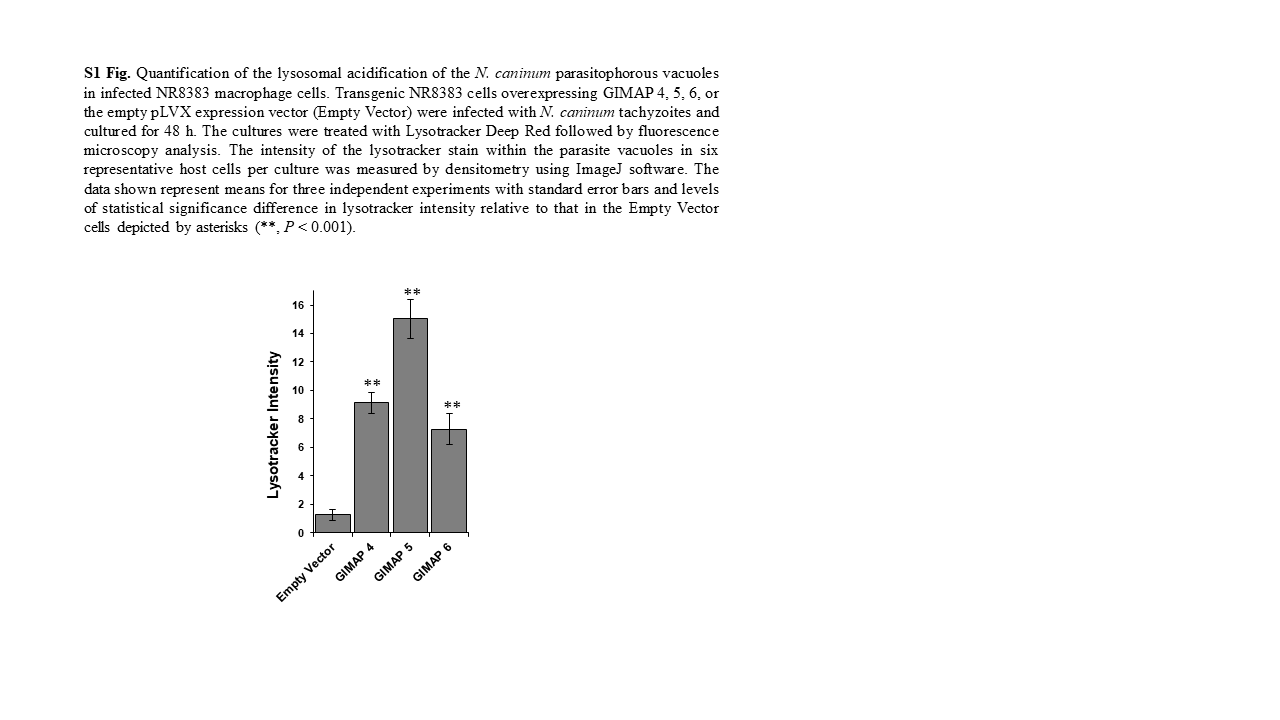

Supplement: Supplementary file 1 [file Image1.tif]

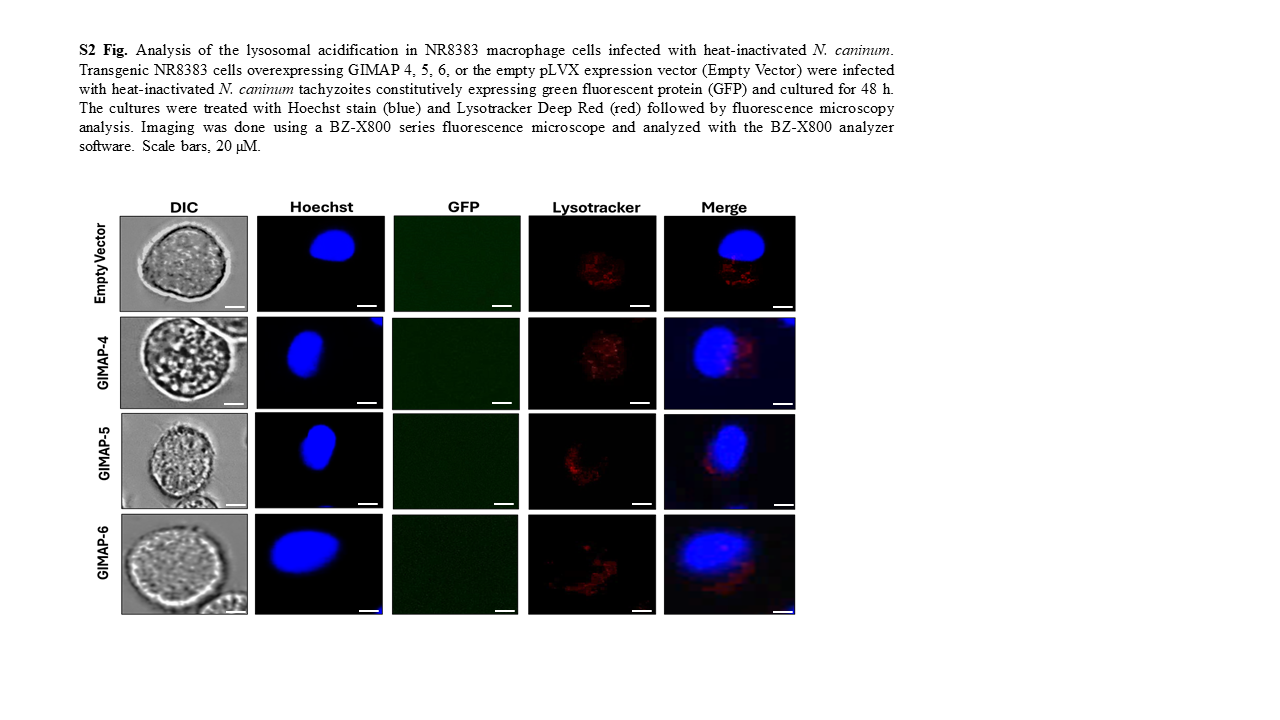

Supplement: Supplementary file 2 [file Image2.tif]

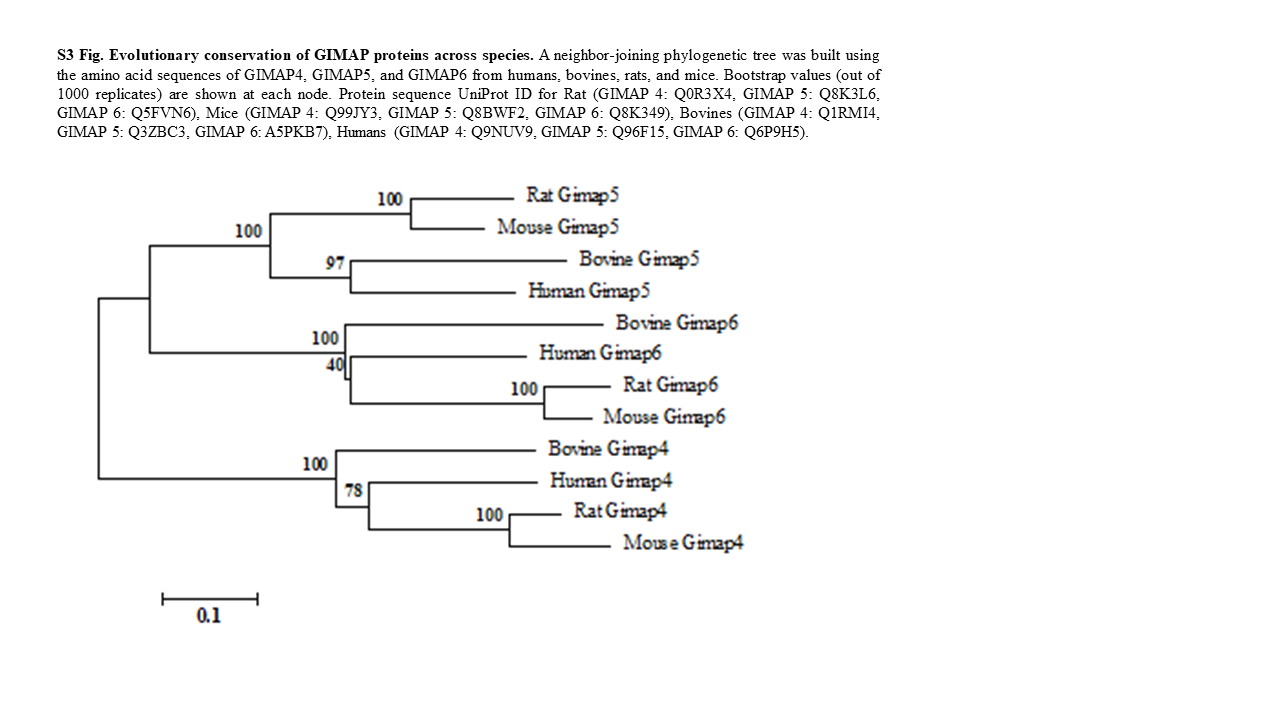

Supplement: Supplementary file 3 [file Image3.tif]
